# Impact of Maternal Micronutrient Intake on Gestational Diabetes Risk: Results from Greece’s BORN2020 Prospective Cohort Study

**DOI:** 10.3390/nu16091375

**Published:** 2024-04-30

**Authors:** Antigoni Tranidou, Emmanuella Magriplis, Aikaterini Apostolopoulou, Ioannis Tsakiridis, Violeta Chroni, Eirini Tsekitsidi, Ioustini Kalaitzopoulou, Nikolaos Pazaras, Michail Chourdakis, Themistoklis Dagklis

**Affiliations:** 1 3rd Department of Obstetrics and Gynecology, School of Medicine, Faculty of Health Sciences, Aristotle University of Thessaloniki, 541 24 Thessaloniki, Greece; antigoni.tranidou@gmail.com (A.T.); iotsakir@gmail.com (I.T.); 2 Laboratory of Hygiene, Social & Preventive Medicine and Medical Statistics, School of Medicine, Faculty of Health Sciences, Aristotle University of Thessaloniki, 541 24 Thessaloniki, Greece; katapost@yahoo.gr (A.A.); violetac@auth.gr (V.C.); eirhnh-2@hotmail.com (E.T.); ioustinik@auth.gr (I.K.); nikospaz@hotmail.com (N.P.);; 3 Department of Food Science and Human Nutrition, Agricultural University of Athens, Iera Odos 75, 118 55 Athens, Greece; emagriplis@eatsmart.gr

**Keywords:** maternal, nutritional intake, pregnancy, gestational diabetes mellitus (GDM), dietary reference values (DRVs), EFSA, Mediterranean diet (MD)

## Abstract

Understanding how maternal micronutrient intake and dietary habits impact gestational diabetes mellitus (GDM) is crucial. Data from 797 pregnant women were prospectively analyzed to assess GDM status with the oral glucose tolerance test (OGTT). Nutritional intake was evaluated using a validated food frequency questionnaire (FFQ) across two periods: Period A, covering 6 months before pregnancy, and Period B, from pregnancy onset to mid-gestation (24 weeks). Micronutrient intakes were compared against the European Food Safety Authority (EFSA) dietary reference values (DRVs) and were used to estimate the mean adequacy ratio (MAR) to assess dietary adequacy. GDM was diagnosed in 14.7% (*n* = 117) of women with the characteristics of a higher mean maternal age (MA) and pre-pregnancy body mass index (BMI). Out of the 13 vitamins assessed, biotin, folate, niacin, and pantothenic acid were found significantly higher in the GDM group, as did iron, magnesium, manganese, phosphorus, and zinc from the 10 minerals. The results were influenced by the timing of the assessment. Importantly, MAR was higher during pregnancy and was found to increase the risk of GDM by 1% (95%CI: 1, 1.02). A sensitivity analysis revealed that reducing MAR significantly raised the GDM risk by 68% (95%CI: 1.02, 2.79). No association was revealed between adherence to the Mediterranean diet (MD) and GDM risk. These findings highlight areas for further investigation into whether dietary modifications involving these specific micronutrients could effectively influence GDM outcomes.

## 1. Introduction

Gestational diabetes mellitus (GDM) refers to a carbohydrate intolerance that manifests as hyperglycemia of varying severity and initiates or is first observed during pregnancy [1]. This condition, exacerbated by factors such as genetic predisposition, hormonal changes in pregnancy that increase insulin resistance, and lifestyle factors including diet and physical activity, leads to varied severities of hyperglycemia [2]. GDM poses both immediate and long-term risks to maternal and fetal health, potentially leading to outcomes such as pre-eclampsia, type 2 diabetes mellitus (T2DM), and macrosomia [3,4,5]. The pathophysiological changes that occur in normal pregnancy predispose the mother and the fetus to a potentially diabetogenic state [6], highlighting the importance of early diagnosis and efficient management [7,8].

The role of diet is particularly significant, as nutritional status directly influences the risk and management of GDM [9,10] with research suggesting that diets of low nutritive value, during pregnancy, including minerals and vitamins, are associated with an increased risk of GDM, while diets rich in fruits, vegetables, whole grains, and lean proteins are linked to a lower risk of GDM [11,12], including the Mediterranean diet (MD), which is renowned for its health benefits and its potential to reduce GDM risk, which ranges from 39% [13] to 80% with high adherence levels before and during pregnancy [14].

Studies also highlight the critical roles of vitamin D [15] and magnesium in women with GDM, improving glycemic control and maintaining adequate nutrition during pregnancy [16]. Furthermore, certain dietary components before pregnancy, such as a high intake of heme iron, have been associated with increased GDM risk and it is important to note that both iron and other nutrients such us iron and vitamin D and folate play significant roles. While vitamin D is commonly known as the ‘sunshine vitamin’ due to its synthesis through skin exposure to sunlight, dietary sources and supplementation of vitamin D also significantly contribute to maintaining adequate levels. This is especially pertinent for pregnant women at high risk for GDM, who may have insufficient dietary intakes of vitamin D and folate, necessitating careful consideration of dietary adjustments and supplementation to meet their nutritional needs [17,18].

Despite the growing interest and ongoing research into how diet influences GDM, further studies are required to fully understand this relationship. The current research suggests a need for a comprehensive view that not only focuses on isolated nutrients but also their combined effects. This approach is vital, as evidenced by a study indicating that higher overall micronutrient adequacy before pregnancy, assessed through the mean adequacy ratio (MAR), correlated with a lower GDM risk [19]. In addition, it has been underscored that physical activity as a modifiable risk factor, when appropriately managed, demonstrates a dose–response relationship with the risk of GDM; higher levels of physical activity in both the first and second trimesters are associated with reduced odds for GDM, with this relationship being stronger in the first trimester [20]. This can help mitigate the risk of GDM by improving insulin sensitivity and managing weight gain during pregnancy [21].

Therefore, the objectives of the current study were (i) to estimate micronutrient adequacies in a Greek pregnant cohort regarding the micronutrient intakes of pregnant women in Northern Greece, relative to GDM risk both prior and also during pregnancy, while also adjusting for energy intake and physical activity and other relevant confounding factors, (ii) to assess these intakes against pre-pregnancy and pregnancy-related dietary recommendations, as indicated by the European Food Safety Authority (EFSA) guidelines, (iii) to assess overall dietary adequacy using MAR in association with GDM risk, and (iv) to evaluate whether adherence to MD had an effect on GDM risk in this Greek pregnant cohort.

## 2. Materials and Methods

### 2.1. Study Population

The current study is part of BORN2020, a large prospective population-based cohort study that commenced in Thessaloniki in 2020, with the aim of collecting and analyzing data on the physical and nutritional status of pregnant women in Northern Greece. Women enrolled at their first planned visit for their routine care check, during weeks 11^+0^–13^+6^ of gestational age, at the 3rd Department of Obstetrics and Gynecology Clinic, Aristotle University, Thessaloniki, Greece, between January 2020 and January 2024. The Bioethics Committee of Aristotle University of Thessaloniki granted their approval (5/12/April 2022). All women were informed about the study, and, upon agreement for participation, written consent was obtained.

The following criteria precluded participation in the study: (i) age < 18 years, (ii) serious medical condition (e.g., renal disease), (iii) adhering to particular dietary trends (e.g., vegan or vegetarian), (iv) pre-existing diabetes prior to GDM diagnosis (e.g., type 1 diabetes mellitus, T2DM), (v) miscarriage or termination before 28 weeks of gestation to ensure screening for GDM, (vi) missing data including health metrics, dietary intakes, or other variables crucial to the analyses.

### 2.2. Outcome Measures

Maternal characteristics, such as age, parity, gravidity, and past medical history, were recorded. During first perinatal visit, which was between 11^+0^ and 13^+6^ weeks of gestation, the following anthropometric features were recorded: weight was measured in kilograms (kg) using a standard digital scale, and height was measured with a stadiometer in centimeters (cm). Pre-pregnancy body mass index (BMI) was computed according to BMI classification standards [22].

### 2.3. GDM Assessment

The guidelines for the diagnosis of GDM used in this study are the ones recommended by the Hellenic Society of Obstetricians and Gynecologists (HSOG) [23], and are in accordance with the Hyperglycemia and Adverse Pregnancy Outcomes (HAPO) study [24]. All pregnant individuals were administered 75 g per os for the oral glucose tolerance test (OGGT) after having fasted for 8 h and having had a rich carbohydrate diet for three days prior to testing. The diagnosis of GDM was thus established when at least one blood glucose measurement was equal to or higher than the established thresholds: fasting levels ≥ 92 mg/dL, blood glucose ≥ 180 mg/dL after one hour, and blood glucose ≥ 153 mg/dL after two hours.

### 2.4. Dietary Assessment

Dietary assessment was performed using a validated semi-quantitative food frequency questionnaire (FFQ), modified for this specific population, based on earlier FFQs that were created for evaluating diet in Mediterranean communities [25] and included 46 foods and beverages that were important for gestation and frequently consumed in Greece. These were then grouped into 14 food categories [26,27,28]. Women were interviewed by two trained professionals to report frequency of intake as per daily, weekly, or monthly intakes. More specifically the following options were given: (i) x portions daily/per week/per month or (ii) never. Portion sizes were provided as household units (e.g., cups, spoons) and metric values of grams/milliliters in order to provide for an accurate estimate of the actual level of consumption. Household units were converted into edible amounts of food, using standardized scales. Frequency and quantity were finally converted into total grams (or mL) of food (or beverages) per day. All data from the participants were entered into food analysis software for Windows (Nutrisurvey V. 2007) to assess energy and micronutrient intakes for all women. Using the FFQ, data on the nutritional status of the enrollees in the two predefined time periods were collected. Period A referred to the time frame up to six months prior to pregnancy, and Period B referred to the time frame from conception until mid-gestation at 24 weeks. For assessment of micronutrient nutrient intake before gestation, the FFQ was completed by means of an oral interview, conducted by qualified personnel, at the first perinatal routine care visit at 11^+0^–13^+6^ weeks of gestation. Respectively, for the evaluation of the consumption of micronutrients during pregnancy, the FFQ was completed again using the same procedure during mid-gestation, at 24 gestational weeks. Roughly 20 min were allotted for each interview.

The micronutrients that were computed included vitamins (biotin, B12, folate, niacin, pantothenic acid, B2, B1, A, B6, C, D, E, K), minerals (sodium, calcium, potassium, magnesium, phosphorus, zinc, iron, folate), and water. Alcohol consumption from the FFQ was also recorded. For vitamin A, the calculation included all carotenoids, while, for niacin, tryptophan was taken into account, and for vitamin E, all tocopherols were considered.

Information on supplement(s) consumed before and during pregnancy were also collected. Women were specifically asked whether they had been taking any supplements; responses were categorized simply as ‘yes’ if they had taken any supplements and ‘no’ if they had not.

Results from the two FFQs were compared to the recommendations from the EFSA. For time Period A, the micronutrient intakes were compared to recommendations for adult women aged > 18 years old, while the outcomes from time Period B were compared to the suggested references for pregnant populations.

In the case of smokers, due to the additional requirement of 35 mg/day because of the higher metabolic rate, levels of Vitamin C were accordingly compared based on indicated reference values for smokers [29].

### 2.5. Micronutrient Adequacy and Comparison to Recommendations from the EFSA

In order to assess micronutrient adequacy, the reference values suggested by the EFSA were used. The average requirement (AR) cut-point method was applied to compare intakes to the EFSA recommendations. In the case that no AR was available, the average intake (AI) was used as an alternative for the comparison and was used to assess nutrient adequacy.

To measure the adequacy of each micronutrient, the nutrient adequacy ratio (NAR) was calculated by dividing the daily intake to the one suggested by the EFSA. An NAR has a scale from 0 to 1. When its value is 1, this indicates that the intake of the micronutrient is equal to the recommendation; otherwise, values below 1 mean that the intake is lower than the recommendation, namely: inadequate. In order to assess for the overall dietary quality, the MAR was calculated by summing all NAR values and dividing them by the number of the micronutrients that were evaluated. This is an index obtained using the mean of the adequacy ratios for all nutrients in a diet. An MAR of 1 or >1 denotes that the intake is equal to or higher than the recommendation, while an MAR below 1 is indicative of micronutrient inadequacies. Whereas NAR offers a precise measurement for the adequacy of individual nutrients, MAR provides a comprehensive picture of dietary quality by evaluating the sufficiency of numerous nutrients in a meal. These steps can aid both patients and medical professionals in identifying vitamin shortages and altering an individual’s diet appropriately to maximize the potential for optimal health outcomes.

### 2.6. Adherence to Mediterranean Diet

The adherence to MD was assessed using the Mediterranean Diet Score (MDS) developed by Trichopoulou et al. and validated within the Greek population [30].

### 2.7. Statistical Analysis

For the analysis of continuous attributes, in cases of normally distributed variables, the *t*-test was utilized to assess differences with respect to the GDM outcome. In cases of non-normally distributed variables, Mann–Whitney test was utilized. The normality was accessed using the Shapiro–Wilk test. For binary attributes, chi-squared test was used.

With regard to the analysis of micronutrients, the adjusted odds ratio (aOR) was computed using a logistic regression model. The adjustment was made with respect to energy intake, supplement intake, whether assisted reproductive technology (ART) was used to conceive, physical activity (how many hours walking within a week), BMI, age, thyroid status (hypothyroidism, Hashimoto’s disease, hyperthyroidism), smoking status (women were questioned at first perinatal visit whether they had smoked so far, so a yes was considered as smoking during pregnancy, and a no was considered as not being a smoker during pregnancy), and parity status. Additional attributes were computed based on the EFSA recommendations: “above” was above recommendation, indicating that women consumed higher amounts than those recommended by the EFSA. To account for micronutrient inadequacies, the lower level (LL) was calculated, defined as the mean minus 2 standard deviations. Upper-level (UL) toxicities in certain vitamin intakes, as defined by EFSA, were also assessed.

The MAR was computed based on the NARs of the micronutrients. Additionally, a reduced MAR was computed by using a sensitivity analysis and removing micronutrients whose value was above 2 from the population (niacin and vitamin K). The aOR for the continuous MARs was computed using a logistic regression model by adjusting for the same set of attributes presented previously.

For assessing the relationship between MD adherence and GDM risk, the score was categorized into two levels based on the median within the study population. Results for each subgroup regarding MAR and reduced MAR were adjusted for the same attributes as in the analysis of micronutrients.

The R programming language was used to implement all statistics (v4.2.1).

## 3. Results

The current study included 797 pregnant women, with 14.7% being diagnosed with GDM (N = 117). The maternal characteristics of the study population by GDM status are presented in Table 1. The mean maternal age (MA) was higher in the GDM group, and a significant higher proportion of women in the GDM group were over the age of 35 (43.59%) compared to the non-GDM group (27.35%). Pre-pregnancy weight was also significantly higher in the GDM group with a median (interquartile range, IQR) of 65 kg (59–79 kg) for the GDM group and 63 kg (57–73 kg), although height did not differ significantly between the two groups, leading to a significantly higher median BMI before pregnancy (23.7; IQR: 21.7–28.5) compared to the non-GDM group (22.7; IQR: 20.8–26.02), as well. Furthermore, a greater proportion of women in the GDM group had a pre-pregnancy BMI > 30 (21.37%) compared to the non-GDM group (11.03%), with a *p* value of 0.003. Smoking during pregnancy was more prevalent in the GDM group (17.95%) compared to the non-GDM group (8.82%). Gravidity analysis revealed a higher proportion of women with two gestations in the GDM group (42.7%) compared to the non-GDM group (32.35%). No significant differences were found in parity and the proportion of planned pregnancies or conception with assisted reproductive technologies (ARTs), or in the prevalence of thyroid disease, between the two groups.

Micronutrient intakes and comparison to the EFSA guidelines were further divided into two cases: six months prior to pregnancy (Period A), and the first half of pregnancy up until 24 weeks of gestation (Period B). The results for Period A are presented in Supplementary Table S1, and those for Period B are given in Table S2.

### 3.1. Pre-Pregnancy (Period A) and During-Pregnancy (Period B) Nutritional Intakes

Tables S1 and S2 show micronutrient intakes for the period up to six months prior to pregnancy and for the during-pregnancy period. These are found in the Supplementary Material and provide details on the cutoffs used for each micronutrient to determine adequacy/inadequacy. Concerning Period A, intakes did not differ between the two groups (GDM, non-GDM), while, for Period B, several statistically significant differences in micronutrient intakes were identified. Specifically, biotin intake was higher in the GDM group compared to the non-GDM group, with an aOR of 1.03 (95%CI: 1.01–1.05, *p* = 0.002). Similarly, folate, niacin, and pantothenic acid intakes were significantly higher in the GDM population (*p* for all 0.007), and aORs indicated increased intake levels (niacin aOR = 1.07, 95%CI: 1.02–1.13; pantothenic acid aOR = 1.42, 95%CI: 1.1–1.85), while there was no change in the odds associated with folate intake (aOR = 1, 95%CI: 1–1.01).

Moreover, the mineral intakes during this period also showed significant variations, with iron intake being higher in GDM individuals (aOR = 1.19, 95%CI: 1.03–1.39, *p* = 0.018), as well as magnesium (aOR = 1.00, 95%CI: 1–1.01, *p* = 0.008) and manganese (aOR = 1.4, 95%CI: 1.1–1.78, *p* = 0.005), which underscored a potential link to GDM risk. The analysis also revealed a statistically significant difference in phosphorus intake between the groups (*p* = 0.034), but the aOR of 1 (95%CI: 1, 1) indicates that this difference does not represent a meaningful change in the odds of developing GDM. Zinc intake was also associated with a higher consumption observed in the GDM cohort (aOR = 1.16, 95%CI: 1.03–1.31, *p* = 0.012).

### 3.2. NAR and MAR before Pregnancy

For period A, for all nutrients, including biotin, folate, niacin, pantothenic acid, riboflavin (vitamin B2), thiamin (vitamin B1), vitamin A, vitamin B6, vitamin C, vitamin D, vitamin E, and vitamin K, as well as the minerals calcium, copper, iodine, iron, magnesium, manganese, phosphorus, potassium, sodium, and zinc, no significant differences were observed in their NAR values between the GDM and non-GDM groups. This suggests that, for the assessed nutrients, both groups had similar levels of dietary adequacy relative to the EFSA guidelines.

The overall MAR, which provides a composite score of nutrient intake adequacy across the diet, did not significantly differ between the GDM (97.59) and non-GDM (98.36) groups (*p* = 0.7). A similar pattern was observed when considering a reduced MAR, which adjusted for potential overestimations of niacin and vitamin K, with both groups showing comparable scores (GDM: 2.29 vs. non GDM: 2.28, *p* = 0.65). The results are detailed in Table 2.

### 3.3. NAR and MAR during Pregnancy

As noted in Table 3, significant differences were observed in the adequacy of several nutrients between the two groups. The biotin intake adequacy was notably higher in the GDM group (NAR = 0.603) compared to the non-GDM group (NAR = 0.489) and significantly increased GDM risk (aOR = 4.02, 95%CI: 1.66–9.66, *p* = 0.002). Similarly, folate adequacy was significantly higher in the GDM group, but this finding requires caution in interpretation due to the very wide confidence interval, which warrants further investigation (aOR = 91.93, 95%CI: 3.3–2523.79, *p* = 0.007). Niacin and pantothenic acid were also significantly higher in the GDM group (niacin: aOR = 1.99, 95%CI: 1.27–3.13, *p* = 0.002; pantothenic acid: aOR = 5.92, 95%CI: 1.61–21.74, *p* = 0.007).

Minerals such as iron, magnesium, and manganese also showed higher intake adequacy in the GDM group (iron: aOR = 3.54, 95%CI: 1.23–10.13, *p* = 0.018; magnesium: aOR = 7.82, 95%CI: 1.69–35.56, *p* = 0.008; manganese: aOR = 2.77, 95%CI: 1.36–5.66, *p* = 0.005), as did phosphorus (aOR = 1.76, 95%CI: 1.04–2.99, *p* = 0.034) and zinc (aOR = 3.19, 95%CI: 1.28–7.97, *p* = 0.012), highlighting potential differences in mineral intake between women with and without GDM during early gestation.

The overall MAR, which provides a comprehensive view of total micronutrient adequacy, was significantly higher in the GDM group (MAR = 96.27) compared to the non-GDM group (MAR = 92.26), with a *p* value of 0.024. This was also reflected in the reduced MAR, indicating a generally higher dietary adequacy among those diagnosed with GDM (*p* = 0.041).

### 3.4. Comparison with ESFA Guidelines

In Supplementary Material Table S3, the comparison of micronutrient intakes to the EFSA guidelines for Periods A and B is depicted. During Period A, there were no significant deviations from the EFSA recommendations that could be linked with a substantial increase in GDM risk. These results suggest that, overall, there was no clear pattern of excessive intake of these nutrients among the study population in Period A.

Moving to Period B, the analysis revealed a few nutrients whose intake levels were significantly above the EFSA recommendations among the GDM group compared to the non-GDM group. Manganese had an aOR of 2.52 (95%CI: 1.34, 4.6, *p* = 0.003), indicating a higher likelihood of exceeding the recommended intake levels. Zinc also showed a higher probability of intake above the recommended levels, with an aOR of 1.84 (95%CI: 1.09, 3.19, *p* = 0.024), suggesting a distinct difference in the consumption patterns of these minerals during early gestation between the two groups.

### 3.5. Inadequacies Assessment and Toxicity Intakes

To assess micronutrient inadequacies, intakes below 2SD were computed for the two predefined time periods, Period A and Period B. Additionally, to assess possible levels of toxicity, intakes were compared to the upper level (UL) where applicable. For both time periods, no reported intake was found below 2SD or above the UL for the GDM and non-GDM groups. The results of the analysis are presented in Supplementary Tables S4 and S5.

For most vitamins, the proportion of individuals with intakes below 2SDs was minimal, indicating a low prevalence of significant nutritional deficiencies in both groups. Specifically, minor differences were observed in the incidence of low intakes for vitamins such as cobalamin (vitamin B12), folate, pantothenic acid, riboflavin (vitamin B2), thiamin (vitamin B1), vitamin A, and vitamin B6, with no significant disparities between the GDM and non-GDM groups. This suggests that severe deficiencies in these vitamins were not a common issue among the study participants during early gestation.

Similarly, for minerals like calcium, copper, iodine, iron, magnesium, phosphorus, potassium, sodium, and zinc, instances of intake falling below 2SDs were also relatively rare, with no significant differences between the GDM and non-GDM groups. This further supports the notion that substantial mineral deficiencies were not widespread among the cohort.

Regarding intakes exceeding the EFSA’s UL, our study found that such occurrences were generally uncommon for both vitamins and minerals, with no reported instances where the upper intake levels were surpassed. This indicates adherence to safe intake levels and suggests that the risk of nutrient toxicity was low among pregnant women in the study, regardless of GDM status.

For magnesium, a higher proportion of the GDM group had intakes above the UL compared to the non-GDM group (22.2% vs. 12.9%), approaching statistical significance (*p* = 0.066). This suggests a potential area of concern for excessive magnesium intake among women with GDM, warranting further attention and dietary guidance.

In Supplementary Material, Figures S1–S4 provide graphical visualization of intakes and group differences in relation to the EFSA recommendations.

### 3.6. Adherence to Mediterranean Diet

Adherence levels to MDS and GDM risk before and during pregnancy, as assessed for MAR and MAR reduced, revealed no significant associations between MD adherence and GDM risk for either period. The results are presented in the Supplementary Material, in Table S6.

## 4. Discussion

The current study documented an incidence rate of 14.7% in the investigation of GDM. Pre-existing characteristics that were correlated with a reduced risk of GDM were a lower body weight and BMI. On the other hand, being an older mother, especially beyond the age of 35, and smoking were associated with higher chances of developing GDM. The dietary patterns, evaluated using NAR, did not show any significant variations between the GDM and non-GDM groups six months before pregnancy. This finding was supported by the MAR, implying comparable overall nutritional adequacy.

In contrast, there were significant differences in the adequacy of nutritional intake between the two groups during the first half of pregnancy. Significantly, the GDM group exhibited a higher biotin intake, indicating a potential correlation between elevated biotin consumption and an augmented likelihood of GDM. Higher levels of folate, niacin, and pantothenic acid were found in the GDM group, suggesting that these nutrients may have a possible impact during early pregnancy. Mineral assessment showed a significant relationship between GDM risk and iron, magnesium, manganese, phosphorus, and zinc. However, the aORs for folate and phosphorus indicated that they had a negligible effect on GDM risk. The variations in maternal adipose tissue during the initial stages of pregnancy highlight the possible hazards linked to excessive food consumption in relation to GDM. Period B showed a notable excess consumption of nutrients, including manganese and zinc, among the GDM group. This suggests that there may be changes in dietary habits during early pregnancy that could contribute to the development of GDM. While there were no significant abnormalities observed in micronutrient intakes below two standard deviations or over the UL according to the standards set by the EFSA, it is nonetheless pertinent to highlight the trend towards excessive nutrient consumption, particularly for magnesium, in the GDM group. This detailed investigation into MAR and NAR provides a more profound comprehension of how dietary choices might impact the risk of GDM, emphasizing the crucial importance of maintaining a well-rounded nutritional intake throughout pregnancy.

While the overall nutrient intake is generally sufficient, as observed from our findings, certain nutrients are consumed in amounts significantly exceeding the norm in the GDM group, as indicated for Period B. This could reflect a compensatory response to the physiological changes and increased metabolic demands of pregnancy, as women may intentionally or unintentionally consume more foods, including fortified ones. Moreover, while these nutrients are essential for metabolic health, a higher intake may adversely impact glucose metabolism and insulin sensitivity. This highlights the potential for targeted dietary interventions to moderate the intake of specific micronutrients, such as biotin, folate, niacin, and pantothenic acid, for pregnant women at risk of, or managing, GDM.

While the assessment using NAR and MAR analyses revealed certain nutrients associated with increased GDM risk, suggesting potential areas of excess, the detailed inadequacy data paint a more complex picture. For most nutrients, outright inadequacies or excesses as indicated by UL were not prevalent, implying that the increased GDM risk associated with higher intakes of specific nutrients like biotin or folate may not stem from widespread nutrient imbalances but could be related to individual dietary patterns or other metabolic factors. This further underscores the need for personalized dietary guidance focusing on moderation and balance, particularly for nutrients identified as risk factors for GDM.

Evidence suggests that a higher adherence to the Mediterranean diet favors glucose tolerance and reduces GDM risk even in women without GDM [31]. In the analysis of the current study, for both the period before pregnancy and during pregnancy, no association was found between adherence to the MD and GDM risk. More research is needed to fully understand these relationships.

Moreover, in line with our results, a study by Looman et al. highlighted the protective benefits of a balanced diet before pregnancy, characterized by micronutrient adequacy, against GDM risk [19]. Extending the exploration of these benefits during pregnancy, our study highlighted the critical need for personalized nutritional advice for women in pregnancy.

Our research indicated that elevated folate levels during pregnancy are associated with a higher GDM risk, consistent with findings from other studies [32]. This finding aligns with other research indicating that elevated maternal folate, assessed through blood plasma, alongside vitamin B12 insufficiency, may elevate GDM risk [33]. Despite us not observing low vitamin B12 levels in our participants, the association between higher folate levels and GDM risk was clear.

Moreover, in our cohort, iron intake during pregnancy was associated with an increased GDM risk. This finding aligns with findings from other studies, where a higher consumption of heme iron was also associated with increased GDM risk [17,34].

In addition, our findings showed that a higher zinc intake was associated with increased GDM odds. While some studies suggest that GDM is related to an imbalance in maternal serum zinc, indicating that pregnant women may benefit from zinc supplementation, the relationship between serum zinc levels and the pathogenesis of GDM remains controversial. Some studies have reported decreased serum zinc levels in GDM cases compared to non-GDM pregnant women, whereas others have found no significant difference. This contrast within the existing literature highlights the need for further investigation into the nuanced role of zinc in gestational diabetes risk [35].

The magnesium intake during pregnancy in the present cohort was also associated with increased GDM risk. The results of a recent meta-analysis showed that magnesium levels were lower in GDM pregnancies, but this association was more pronounced during the third trimester of pregnancy and observed in case–control studies, while, in cross-sectional studies, there was no such association identified, highlighting the need for a prospective research design to evaluate this relationship [36].

In addition to the above, higher pantothenic acid levels during pregnancy were also associated with GDM risk. A study by Tian et al. that investigated the role of certain metabolomics and GDM risk also identified that, in GDM women, pantothenic acid levels were significantly higher [37].

Increased manganese levels during pregnancy were identified as a risk factor for GDM occurrence. The results from a recent meta-analysis suggested that higher manganese levels may be a risk factor for developing GDM [38].

Biotin intake during pregnancy was also associated with higher GDM odds. We only identified one study other than ours that assessed biotin in serum and GDM risk; however, the findings revealed no association [39]. More studies are needed in order to assess a clear relationship.

Regarding our findings for niacin and phosphorus, we identified no study assessing these micronutrient and their risk in GDM. This indicates a gap in understanding the relationship between these nutrients and their effect on GDM occurrence; hence, more studies are needed in order to reveal potential associations.

In discussing the strengths and limitations of our study, it is important to highlight the comprehensive analysis conducted on an extensive range of micronutrients, further bolstered by adjustments for confounding factors. This approach significantly adds to the study’s value. In addition, all women were screened for GDM from a single care unit, using the same diagnostic criteria, so that diagnosis consistency would be ensured. One of the highlights of the study is that it assessed the dietary intake of the population under study both before and during pregnancy to consider for potential causal links. Additionally, as far as we are aware, the presented analysis is the most extensive investigation regarding the associations between dietary habits and their relation to GDM conducted in Greece. In our analysis, we also calculated the MAR to assess overall micronutrient adequacy, a critical step for evaluating the nutritional quality of diets. This calculation is essential for understanding how well diets meet the nutritional needs essential for reducing GDM risk, beyond just individual nutrient analysis. We adjusted for energy intake to accurately assess micronutrient intake density. This adjustment is crucial as it helps to differentiate the effects on GDM risk of total caloric intake from those of specific nutrients, ensuring that our assessment of micronutrient adequacy reflects true dietary quality without being confounded by variations in energy consumption. In addition, we also adjusted for supplementation to have a more reflective view of the given dietary intake and its risk in GDM.

One of the limitations of the study was the small number of GDM events, which could lead to larger ambiguity, and potentially obscure detectable differences that a larger sample might reveal. Analysis with more GDM events and, generally, the recruitment of more women for analysis is being left for a future work. Another limitation is the potential for recall bias when using an FFQ, due to inaccurate recollections of food consumption. To mitigate this, semi-quantitative FFQs validated via 2 24 h recalls were used to minimize misreporting. An FFQ is a nutritional assessment tool to capture usual long-term intakes. Absolute micronutrient values cannot be determined through any of the nutritional assessment tools, but they are useful to distinguish between individual variations, i.e., higher consumers vs. lower consumers.

These findings suggest that women with GDM may have a slightly better overall nutrient intake adequacy during the first half of gestation compared to those without GDM, particularly for certain vitamins and minerals. However, micronutrient intake alone may not suffice to prevent and/or regulate GDM onset. This was investigated because supplements are prescribed regularly during gestation but dietary assessment is often neglected. Dietary intake and foods contributing to nutrient adequacy may be more informative. To ensure a more accurate evaluation of micronutrient intake, all results were adjusted for energy intake. Building on this approach, the primary aim of the study was to assess micronutrient adequacy, specifically, since many supplements are provided during this life stage. The outcome emphasizes that this may not suffice, and in-depth assessment of diet quality in relation to micronutrient status should be assessed, especially in an era where food fortification has become a trend. The clinical significance of these differences and their impact on GDM management and their outcomes require further investigation. Although the current study adjusted for numerous confounders, consideration of the effect of additional lifestyle determinants and genetic factors that contribute to GDM will further aid in understanding the potential for dietary modifications in the prevention and management of GDM.

## 5. Conclusions

Maternal nutrition before and during pregnancy should not be underestimated. The micronutrient intakes of several micronutrients may influence the risk for GDM development, and, thus, more research towards achieving more favorable outcomes against GDM development should be conducted. Furthermore, the current research highlights the need for suggesting preventing guidelines, but many studies are needed in order to have a better understanding of this interplay between maternal nutrition and risk for GDM. Hence, healthcare professionals should prioritize the assessment of maternal nutritional status and provide appropriate guidance to women regarding a balanced and nutrient-rich diet to optimize maternal and fetal health outcomes. Moreover, due to the variability of the current evidence, the extent to which these intakes relate to different population subgroups is difficult to quantify. Large-scale ongoing and high-quality studies during the preconception and pregnancy periods, like the one presented in this paper, need to be designed in other geographical areas, as well, to ensure informed decisions.

## Figures and Tables

**Table 1:** Comparison of maternal characteristics between the GDM and non-GDM groups (total N = 797). Descriptive statistics: mean (SD), percentiles, or proportion.

	GDM (117)	Non-GDM (680)	*p*-Value	Comparison Result
MA (mean, SD)	34.15 (±4.48)	32.1 (±4.89)	*p* < 0.0001 ***	↑ in GDM
MA > 35 (n %)	51 (43.59%)	186 (27.35%)	*p* < 0.001 ***	↑ in GDM
Wt before pregnancy (median (IQR))	65 (59, 79)	63 (57, 73)	0.008 **	↑ in GDM
Ht (centimeters)	165 (161, 170)	165 (162, 170)	0.27	-
BMI before pregnancy (median (IQR))	23.7 (21.7, 28.5)	22.7 (20.8, 26.02)	0.002 **	↑ in GDM
BMI > 25 before pregnancy (median (IQR))	46 (39.32%)	214 (31.47%)	0.12	-
BMI > 30 before pregnancy (median (IQR))	25 (21.37%)	75 (11.03%)	0.003 **	↑ in GDM
Smoking during pregnancy	21 (17.95%)	60 (8.82%)	0.004 **	↑ in GDM
Thyroid disease	13 (11.11%)	93 (13.68%)	0.54	-
Gravidity (n%)	77 (65.81%)	403 (59.26%)	0.22	-
0	40 (34.19%)	277 (40.74%)	0.22	-
1	50 (42.74%)	220 (32.35%)	0.037 *	↑ in GDM
2	17 (14.53%)	121 (17.79%)	0.47	-
3	7 (5.98%)	40 (5.88%)	0.87	-
4	3 (2.56%)	17 (2.5%)	0.78	-
5	0 (0%)	4 (0.588%)	-	-
6	0 (0%)	1 (0.147%)	-	-
Parity				
0	60 (51.28%)	347 (51.03%)	0.96	-
1	44 (37.61%)	253 (37.21%)	0.98	-
2	12 (10.26%)	69 (10.15%)	0.9	-
3	1 (0.855%)	9 (1.32%)	0.98	-
4	0 (0%)	2 (0.294%)	-	-
Planned pregnancy (n%)	103 (88.03%)	582 (85.71%)	0.6	-
Conception with ART (n%)	11 (9.4%)	48 (7.06%)	0.48	-

IQR: interquartile range, percentiles (25th, median, 75th); n: number of participants; SD: standard deviation; MA: maternal age in years; BMI: body mass index in kilograms divided by height in square meters (kg/m^2^). Thyroid disease includes hypothyroidism, hyperthyroidism, and Hashimoto’s disease. Conception ART: conception with assisted reproductive technologies; the “↑” indicates that the metric for the GDM group is higher compared to the non-GDM group; “*” indicates *p* value < 0.05, “**” indicates *p* value < 0.01, “***” indicates *p* value < 0.001; Statistics of the population for the different attributes: For normally distributed variables, the mean and the SD are presented; otherwise, the median and quartiles are shown. Normality was accessed using the Shapiro–Wilk test. The *p*-value was computed using *t*-test (normally distributed) or Mann–Whitney (non-normally distributed variables) for continuous attributes, or chi-squared test for binary attributes.

**Table 2:** Nutrient adequacy ratio (NAR) and mean adequacy ratio (MAR) among individuals with GDM and non-GDM 6 months prior to gestation.

	Intake 6 Months Prior to Pregnancy (Period A)			
	GDM (N = 117)	Non-GDM (N = 680)	*p* value	aOR
	NAR	NAR		
	Median (25, 75 percentiles)	Median (25, 75 percentiles)		
Vitamins				
Biotin	0.513 (0.38, 0.706)	0.493 (0.376, 0.651)	1	0.99 (0.41, 2.38)
Cobalamin (Vitamin B12)	0.826 (0.649, 1.07)	0.774 (0.583, 0.997)	0.83	1.06 (0.58, 1.94)
Folate	0.585 (0.471, 0.744)	0.575 (0.481, 0.685)	0.41	0.63 (0.18, 1.84)
Niacin	2.06 (1.78, 2.4)	2.03 (1.75, 2.33)	0.48	1.16 (0.76, 1.77)
Pantothenic acid	0.614 (0.518, 0.78)	0.596 (0.497, 0.721)	0.85	0.88 (0.25, 2.95)
Riboflavin (Vitamin B2)	0.688 (0.485, 0.859)	0.631 (0.478, 0.799)	0.74	0.87 (0.39, 1.78)
Thiamin (Vitamin B1)	1.1 (0.892, 1.3)	1.15 (0.947, 1.34)	0.19	0.62 (0.3, 1.23)
Vitamin A	1.66 (1.28, 2.02)	1.64 (1.33, 1.97)	0.33	0.81 (0.53, 1.22)
Vitamin B6	0.759 (0.632, 0.911)	0.765 (0.642, 0.909)	0.23	0.48 (0.14, 1.57)
Vitamin C	1.02 (0.787, 1.26)	1.04 (0.821, 1.27)	0.17	0.7 (0.41, 1.15)
Vitamin D	0.062 (0.035, 0.098)	0.061 (0.036, 0.103)	0.83	0.69 (0.01, 19.11)
Vitamin E	0.881 (0.668, 1.07)	0.797 (0.639, 1.01)	0.41	1.23 (0.7, 2.01)
Vitamin K as phylloquinone	2.32 (1.86, 2.91)	2.29 (1.93, 2.71)	0.55	0.89 (0.6, 1.28)
Minerals				
Calcium	1.03 (0.728, 1.22)	0.936 (0.704, 1.19)	0.56	0.86 (0.5, 1.37)
Copper	1.26 (0.981, 1.51)	1.2 (1.02, 1.43)	1	0.99 (0.48, 2.04)
Iodine	0.646 (0.518, 0.751)	0.621 (0.511, 0.76)	0.36	0.6 (0.2, 1.71)
Iron	0.987 (0.839, 1.16)	0.969 (0.823, 1.15)	0.98	1 (0.43, 2.35)
Magnesium	0.647 (0.548, 0.777)	0.624 (0.536, 0.742)	0.96	1.04 (0.22, 4.74)
Manganese	0.574 (0.407, 0.794)	0.518 (0.392, 0.713)	0.26	1.55 (0.71, 3.34)
Phosphorus	1.57 (1.27, 1.89)	1.46 (1.17, 1.78)	0.97	0.98 (0.6, 1.56)
Potassium	0.509 (0.448, 0.613)	0.508 (0.428, 0.603)	0.57	0.61 (0.11, 3.28)
Sodium	1.07 (0.77, 1.27)	1 (0.811, 1.22)	0.71	0.87 (0.42, 1.75)
Zinc	1.42 (1.21, 1.65)	1.31 (1.12, 1.54)	0.57	1.18 (0.66, 2.22)
MAR	97.59 (87.78, 116.09)	98.36 (84.6, 110.72)	0.7	0.99 (0.98, 1)
MAR reduced	2.29 (2.03, 2.75)	2.28 (1.94, 2.61)	0.65	0.9 (0.58, 1.39)

NAR: nutrient adequacy ratio; MAR: mean adequacy ratio. The adjusted odds ratio (aOR) was computed using a logistic regression model. The adjustment was made with respect to energy, ART, supplement intake, physical activity, BMI, maternal age, thyroid status, smoking status, and parity status.

**Table 3:** Nutrient adequacy ratio (NAR) and mean adequacy ratio among individuals with GDM and non-GDM individuals for first half of gestation.

	Intake for First Half of Gestation (Period B)			
	GDM (N = 117)	Non-GDM (N = 680)		
	NAR	NAR		
	Median (25, 75 percentiles)	Median (25, 75 percentiles)	*p* value	aOR
Vitamins				
Biotin	0.603 (0.412, 0.756)	0.489 (0.384, 0.638)	0.56	1.16 (0.68, 1.93)
Cobalamin (Vitamin B12)	0.708 (0.578, 0.922)	0.691 (0.525, 0.883)	0.48	1.35 (0.57, 3.17)
Folate	0.265 (0.21, 0.334)	0.243 (0.2, 0.294)	0.64	0.72 (0.17, 2.75)
Niacin	2.06 (1.74, 2.45)	1.99 (1.7, 2.25)	0.018 *	3.54 (1.23, 10.13)
Pantothenic acid	0.643 (0.532, 0.798)	0.599 (0.497, 0.728)	0.008 **	7.82 (1.69, 35.56)
Riboflavin (Vitamin B2)	0.595 (0.459, 0.744)	0.528 (0.407, 0.682)	0.005 **	2.77 (1.36, 5.66)
Thiamin (Vitamin B1)	1.07 (0.902, 1.43)	1.11 (0.908, 1.33)	0.034 *	1.76 (1.04, 2.99)
Vitamin A	1.53 (1.31, 1.89)	1.51 (1.26, 1.8)	0.11	4.19 (0.71, 24.27)
Vitamin B6	0.681 (0.541, 0.807)	0.656 (0.543, 0.787)	0.54	1.25 (0.6, 2.58)
Vitamin C	1.15 (0.857, 1.52)	1.13 (0.874, 1.42)	0.012 *	3.19 (1.28, 7.97)
Vitamin D	0.057 (0.035, 0.098)	0.058 (0.034, 0.096)	0.56	1.16 (0.68, 1.93)
Vitamin E	0.849 (0.671, 1.11)	0.798 (0.632, 1.03)	0.48	1.35 (0.57, 3.17)
Vitamin K as phylloquinone	2.47 (2.04, 3)	2.33 (1.95, 2.75)	0.64	0.72 (0.17, 2.75)
Minerals				
Calcium	1.07 (0.767, 1.24)	0.942 (0.71, 1.21)	0.56	1.16 (0.68, 1.93)
Copper	1.18 (0.964, 1.31)	1.1 (0.916, 1.3)	0.48	1.35 (0.57, 3.17)
Iodine	0.491 (0.438, 0.59)	0.475 (0.401, 0.572)	0.64	0.72 (0.17, 2.75)
Iron	0.975 (0.805, 1.22)	0.956 (0.804, 1.12)	0.018 *	3.54 (1.23, 10.13)
Magnesium	0.665 (0.547, 0.824)	0.623 (0.526, 0.747)	0.008 **	7.82 (1.69, 35.56)
Manganese	0.574 (0.431, 0.84)	0.518 (0.396, 0.722)	0.005 **	2.77 (1.36, 5.66)
Phosphorus	1.59 (1.33, 1.95)	1.49 (1.17, 1.8)	0.034 *	1.76 (1.04, 2.99)
Potassium	0.527 (0.421, 0.637)	0.499 (0.418, 0.6)	0.11	4.19 (0.71, 24.27)
Sodium	1.03 (0.824, 1.26)	1.01 (0.83, 1.24)	0.54	1.25 (0.6, 2.58)
Zinc	1.2 (1.03, 1.36)	1.11 (0.951, 1.3)	0.012 *	3.19 (1.28, 7.97)
MAR	96.27 (82.9, 113.67)	92.26 (80.02, 106.39)	0.024 *	1.01 (1, 1.02)
MAR reduced	2.2 (1.86, 2.65)	2.12 (1.83, 2.48)	0.041 *	1.68 (1.02, 2.79)

NAR: nutrient adequacy ratio; MAR: mean adequacy ratio; “*” indicates *p* value < 0.05, “**” indicates *p* value < 0.01, The adjusted odds ratio (aOR) was computed using a logistic regression model. The adjustment was made with respect to energy, ART, supplement intake, physical activity, BMI, maternal age, thyroid status, smoking status, and parity status.

## Data Availability

Due to privacy and confidentiality considerations, the data supporting the findings of this study are not publicly available.

## Supplementary Materials

The following supporting information can be downloaded at https://www.mdpi.com/article/10.3390/nu16091375/s1, Table S1: Micronutrient intakes and dietary reference values by the European Food and Safety Authorities (EFSA) for adult women > 18 years old; Table S2: Micronutrient intakes and dietary reference values by the European Food and Safety Authorities (EFSA) for pregnant women; Table S3: Comparison of micronutrient intakes to EFSA guidelines for Period A (up to six months prior to gestation) and Period B (until first half of gestation); Table S4: Intakes of micronutrients below 2SDs or above the upper level (UL) as suggested by the European Food and Safety Authority (EFSA) for non-pregnant women at six months prior to pregnancy; Table S5: Intakes of micronutrients below 2SDs or above the upper level (UL) as suggested by the European Food and Safety Authority (EFSA) for pregnant women for the first half of gestation; Table S6: Adherence to MDS and risk for GDM before and during pregnancy for the Mean adequacy ratio (MAR); Figure S1: Vitamin intakes above EFSA recommendations in GDM and non-GDM groups for up to six months before pregnancy; Figure S2: Mineral and water intakes above EFSA recommendations in GDM and non-GDM groups for up to six months before pregnancy; Figure S3: Vitamin intakes above EFSA recommendations in GDM and non-GDM groups during pregnancy; Figure S4: Mineral and water intakes above EFSA recommendations in GDM and non-GDM groups during pregnancy.
