# Supplementary material for: Impact of Maternal Micronutrient Intake on Gestational Diabetes Risk: Results from Greece’s BORN2020 Prospective Cohort Study
Source: Nutrients. 2024 Apr 30;16(9):1375. doi: 10.3390/nu16091375 (PMC11085310; doi:10.3390/nu16091375)

Supplementary material

Tables

Table S1. Micronutrient intakes and dietary reference values by the European Food and Safety Authorities (EFSA) for adult women >18 years old.

	EFSA reference values for adult women >18 years old	Intake 6 months prior to pregnancy (Period A)						p value	Adjusted odds ratio
		GDM (N=117)			Non GDM (N=680)				
		25th	median	75th	25th	median	75th		
**Vitamins**									
Biotin	AI 40 µg/day	15.21	20.51	28.24	15.03	19.72	26.04	1	0.99 (0.97,1.02)
Cobalamin (Vitamin B12)	AI 4 µg/day	2.6	3.31	4.29	2.33	3.1	3.99	0.83	1.01 (0.87,1.18)
Folate	AR 250 µg DFE/day	117.69	146.3	186	120.3	143.7	171.2	0.41	0.99 (0.99,1)
Niacin	AR 1.3 mg NE/MJ	14.45	17	21.03	13.32	16.3	19.3	0.69	1 (0.96,1.05)
Pantothenic acid	AI 5 mg/day	2.59	3.07	3.9	2.49	2.98	3.06	0.85	0.97 (0.75,1.24)
Riboflavin (Vitamin B2)	AR 1.3 mg/day	0.63	0.9	1.12	0.62	0.82	1.04	0.74	0.9 (0.48,1.56)
Thiamin (Vitamin B1)	AR 0.072 mg/MJ	0.42	0.5	0.63	0.4	0.51	0.61	0.29	0.47 (0.11,1.85)
Vitamin A	AR 490 µg RE/day	627.67	811.08	988.1	651.2	803.92	964.74	0.33	0.99 (0.99,1)
Vitamin B6	AR 1.3 mg/day	0.82	0.99	1.18	0.83	0.99	1.18	0.23	0.56 (0.22,1.41)
Vitamin C	AR 80 mg/day	62.9	81.32	100.9	67.7	83.07	101.8	0.17	0.99 (0.98,1)

	AR* 115mg /day								
Vitamin D	AI 15 µg/day	0.53	0.94	1.47	0.54	0.91	1.55	0.83	0.97 (0.76,1.21)
Vitamin E	AI 11 mg/day	7.35	9.69	11.8	7.03	8.76	11.07	0.41	1.01 (0.96,1.06)
Vitamin K as phylloquine	AI 70 µg/day	130.5	162.5	203.5	135.4	160.6	190	0.55	0.99 (0.99,1)
**Minerals**									
Calcium	AR 750 mg/day	546.12	770.6	918.5	533	702	906.6	0.55	0.99 (0.99,1)
Copper	AI 1.3 mg/day	1.28	1.64	1.96	1.33	1.56	1.86	1	0.99 (0.57,1.73)
Iodine	AI 150 µg/day	77.7	96.9	112.7	76.7	93.1	114	0.36	0.99 (0.98,1)
Iron	AR 7 mg/day	5.87	6.9	8.15	5.76	6.78	8.02	0.98	1 (0.88,1.12)
Magnesium	AI 300 mg/day	164.3	194.1	233.2	160.7	187.3	222.5	0.96	1 (0.99,1)
Manganese	AI 3 mg/day	1.22	1.72	2.38	1.18	1.56	2.14	0.26	1.15 (0.89,1.49)
Phosphorus	AI 550 mg/day	699.1	864.8	1040.9	645.6	804.2	980	0.97	0.99 (0.99,1)
Potassium	AI 3500 mg/day	1568.7	1781.8	2144.2	1499.3	1777.4	2110	0.57	0.99 (0.99,1)
Sodium	Safe and adequate intake: 2g/day	1540.3	2139	2548.8	1621.7	2001.8	2449.4	0.71	0.99 (0.99,1)
Zinc	AI 6.2 mg/day	7.48	8.82	10.2	6.95	8.15	9.56	0.57	1.02 (0.93,1.13)
**Water**	AI 2 L/day	1956.18	2383.99	2972.78	1759.52	2295.97	2850.49	0.41	1 (0.99,1)
**Alcohol**	-	-	0.438	3.76	-	-	1.88	0.21	1.04 (0.96,1.11)

The adjusted Odds Ratio (aOR) was computed using a logistic regression model. The adjustment was made with respect to: energy, supplements intake, ART, physical activity, BMI, maternal age, thyroid status, smoking status, and parity status. AR*: AR vitamin C recommendations for smokers. "-"means not enough data or zero consumption reported. No safe limit for alcohol consumption has been established.

Table S2. Micronutrient intakes and dietary reference values by the European Food and Safety Authorities (EFSA) for pregnant women.

	EFSA for pregnant women	Intake during the first half of pregnancy (Period B)						p value	Adjusted odds ratio
		GDM (N=117)			Non GDM (N=680)				
		25th	median	75th	25th	median	75th		
**Vitamins**									
Biotin	AI 40 µg/day	16.47	24.13	30.2	15.4	19.5	25.5	0.002**	1.03 (1.01,1.05)
Cobalamin (Vitamin B12)	AI 4.5 µg/day	2.6	3.2	4.15	2.36	3.11	4	0.62	1.04 (0.88,1.23)
Folate	AI 600 µg DFE/day	125.8	159.1	200.4	119.8	146.03	176.3	0.007**	1 (1,1.01)
Niacin	AR 1.3 mg NE/MJ	13.7	17.3	20.75	12.7	15.7	18.9	0.007**	1.07 (1.01,1.13)
Pantothenic acid	AI 5 mg/day	2.66	3.21	3.99	2.49	3	3.63	0.007**	1.42 (1.1,1.85)
Riboflavin (Vitamin B2)	AR 1.5 mg/day	0.69	0.89	1.12	0.61	0.79	1.02	0.13	1.63 (0.86,3.09)
Thiamin (Vitamin B1)	AR 0.072 mg/MJ	0.4	0.5	0.63	0.4	0.5	0.6	0.28	2 (0.56,7.08)
Vitamin A	AR 540 µg RE/day	707.4	826.5	1019.6	678.6	813.7	972.5	0.72	1 (0.99,1)
Vitamin B6	AR 1.5 mg/day	0.81	1.02	1.21	0.81	0.98	1.18	0.69	1.19 (0.48,2.88)
Vitamin C	AR 80 mg/day	68.6	92.3	121.5	70	90.2	113.9	0.56	1 (0.99,1)
Vitamin D	AI 15 µg/day	0.53	0.85	1.46	0.51	0.87	1.43	0.54	1.05 (0.87,1.24)
Vitamin E	AI 11 mg/day	7.38	9.34	12.3	7	8.8	11.4	0.16	1.04 (0.97,1.11)
Vitamin K as phylloquine	AI 70 µg/day	143	172.7	210	136.6	162.9	192.5	0.1	1 (0.99,1)
**Minerals**									
Calcium	AR 750 mg/day	575	805.8	916.2	539.3	717.7	911.8	0.59	1 (0.99,1)
Copper	AI 1.5 mg/day	1.45	1.77	1.96	1.37	1.65	1.95	0.48	1.22 (0.69,2.16)
Iodine	AI 200 µg/day	80.23	94.94	114.47	87.63	98.18	118.05	0.64	0.99 (0.99,1)
Iron	AR 7 mg/day	5.64	6.83	8.54	5.63	6.69	7.87	0.018*	1.19 (1.03,1.39)
Magnesium	AI 300 mg/day	164	199.6	247.1	157.8	186.8	224	0.008**	1 (1,1.01)

Manganese	AI 3 mg/day	1.29	1.72	2.52	1.19	1.55	2.17	0.005**	1.4 (1.1,1.78)
Phosphorus	AI 550 mg/day	733.8	871.8	1071.3	642.3	816.8	990.5	0.034*	1 (1,1)
Potassium	AI 3500 mg/day	1474.7	1844.9	2230.9	1464.3	1745.4	2098.5	0.11	1 (0.99,1)
Sodium	Safe and adequate intake: 2 gr/day	1647	2058	2526	1660	2029.3	2481	0.54	1 (0.99,1)
Zinc	AR 7.5 mg/day	7.76	8.98	10.23	7.13	8.31	9.72	0.012*	1.16 (1.03,1.31)
**Water**	AI 2.3 L/day	5	7.14	8	4	6	8	0.64	1 (0.99,1)
**Alcohol**	-	-	-	-	-	-	-	0.21	1.2 (0.88,1.63)

The adjusted Odds Ratio (aOR) was computed using a logistic regression model. The adjustment was made with respect to: energy, ART, supplements intake, physical activity, BMI, maternal age, thyroid status, smoking status, and parity status; "-"means not enough data or zero consumption reported; "*" indicates p value< 0.05, "**" indicates p value <0.01; AR*: AR vitamin C recommendations for smokers. No safe limit for alcohol consumption has been established.

Table S3. Comparison of micronutrient intakes to EFSA guidelines for Period A (up to six months prior to gestation) and Period B (until first half of gestation).

	Above EFSA recommendation for Period A	aOR (95% CI)	p value	Above EFSA recommendation for Period B	aOR (95% CI)	p value
**Vitamins**						
Biotin	AI >40 µg/day			AI >40 µg/day	1.33 (0.48,3.3)	0.55
Cobalamin (Vitamin B12)	AI >4 µg/day	1.06 (0.63,1.74)	0.81	AI >4.5 µg/day	1.16 (0.63,2.05)	0.16
Folate	AR >250 µg DFE/day	1.5 (0.49,4.12)	0.45	AI >600 µg DFE/day	- (-,-)	-
Niacin	AR >1.3 mg NE/MJ	$8.97*10^5$ ($2.37*10^{-30}$,-)	0.98	AR >1.3 mg NE/MJ	$1.56*10^5$ ($6.69*10^{-43}$,-)	0.98
Pantothenic acid	AI >5 mg/day	0.85 (0.28,2.22)	0.76	AI >5 mg/day	1.26 (0.41,3.47)	0.66
Riboflavin (Vitamin B2)	AR >1.3 mg/day	0.98 (0.48,1.86)	0.96	AR >1.5 mg/day	0.76 (0.24,2.05)	0.62
Thiamin (Vitamin B1)	AR >0.072 mg/MJ	0.77 (0.49,1.2)	0.25	AR >0.072 mg/MJ	0.88 (0.57,1.38)	0.6
Vitamin A	AR >490 µg RE/day	0.77 (0.4,1.61)	0.47	AR >540 µg RE/day	0.76 (0.4,1.51)	0.42
Vitamin B6	AR >1.3 mg/day	0.8 (0.41,1.51)	0.52	AR >1.5 mg/day	1.25 (0.49,2.95)	0.61
Vitamin C	AR >80 mg/day AR* >115mg /day	0.83 (0.54,1.28)	0.41	AR >80 mg/day	0.93 (0.6,1.44)	0.75
Vitamin D	AI >15 µg/day	- (-,-)	-	AI >15 µg/day	- (-,-)	-
Vitamin E	AI >11 mg/day	1.17 (0.68,1.99)	0.56	AI >11 mg/day	1.46 (0.87,2.43)	0.15
Vitamin K as phylloquine	AI >70 µg/day	$6.21*10^5$ ($5.98*10^{-19}$,-)	0.98	AI >70 µg/day	1.26 (0.21,24.28)	0.83
**Minerals**						
Calcium	AR >750 mg/day	1.26 (0.81,1.95)	0.3	AR >750 mg/day	1.38 (0.89,2.15)	0.15
Copper	AI	0.61 (0.37,1.01)	0.054	AI >1.5 mg/day	1.19 (0.75,1.93)	0.45

	>1.3 mg/day					
Iodine	AI >150 µg/day	0.86 (0.3,2.12)	0.77	AI >200 µg/day	$5.82*10^{-7}$ (-,$2.81*10^{17}$)	0.98
Iron	AR >7 mg/day	1.12 (0.69,1.8)	0.64	AR >7 mg/day	1.12 (0.68,1.83)	0.65
Magnesium	AI >300 mg/day	1.12 (0.33,3.29)	0.83	AI >300 mg/day	2.74 (0.95,7.51)	0.053
Manganese	AI >3 mg/day	1.5 (0.76,2.83)	0.22	AI >3 mg/day	2.52 (1.34,4.6)	0.003**
Phosphorus	AI >550 mg/day	0.77 (0.41,1.48)	0.42	AI >550 mg/day	1.78 (0.83,4.28)	0.16
Potassium	AI >3500 mg/day	0.76 (0.03,6.73)	0.84	AI >3500 mg/day	1.43 (0.18,7.81)	0.69
Sodium	>2 gr/day	1.22 (0.77,1.93)	0.38	AR >2 gr/d	0.93 (0.59,1.47)	0.77
Zinc	AI >6.2 mg/day	1.68 (0.86,3.56)	0.15	AR >7.5 mg/day	1.84 (1.09,3.19)	0.024*
**Water**	AI >2 L/day	1.36 (0.87,2.18)	0.18	AI >2.3 L/day	1.36 (0.89,2.11)	0.16
**Alcohol**	-	-	-	-	-	-

The adjusted Odds Ratio (aOR) was computed using a logistic regression model. The adjustment was made with respect to: energy, ART, supplements intake, physical activity, BMI, maternal age, thyroid status, smoking status, and parity status; "-"means not enough data or zero consumption reported; "*" indicates p value< 0.05, "**" indicates p value <0.01; AR*: AR vitamin C recommendations for smokers. No safe limit for alcohol consumption has been established.

Table S4. Intakes of micronutrients below 2SDs or above the Upper level (UL) as suggested by the European Food and Safety Authorities (EFSA) for adult non pregnant women.

Micronutrients	6 months prior to pregnancy							
	Intakes below 2SDs				Intakes above the UL			
	GDM	Non GDM	p value	aOR 95% CI	GDM	Non GDM	p value	aOR 95% CI
**Vitamins**								
Biotin	-	3 (0.44)	0.98	$3.82*10^{-6}$(-,$3.73*10^{22}$)	-	-	-	-
Cobalamin (Vitamin B12)	-	-	-	- (-,-)	-	-	-	-
Folate	0 (0%)	4 (0.588%)	0.98	$2.96*10^{-6}$ (-,$9.4*10^{36}$)	-	1 (0.15)	0.98	$8.44*10^{-7}$ (-,$1.34*10^{41}$)
Niacin	0 (0%)	2 (0.294%)	-	$2.73*10^{-6}$ (-,$8.15*10^{36}$)	-	-	-	-
Pantothenic acid	-	4 (0.59)	0.98	$1.3*10^{-6}$ (-,$2.87*10^{29}$)	-	-	-	-
Riboflavin (Vitamin B2)	-	-	-	- (-,-)	--	-	-	-
Thiamin (Vitamin B1)	0 (0%)	2 (0.294%)	1	$2.96*10^{-6}$ (-,$9.4*10^{36}$)	-	-	-	-
Vitamin A	2 (1.71%)	9 (1.32%)	0.39	2.04 (0.29,8.82)	-	(0.15)	0.98	$1.78*10^{-5}$ (-,$4.16*10^{42}$)
Vitamin B6	-	3 (0.44)	0.98	$2.77*10^{-6}$ (-,$1.82*10^{23}$)	-	-	-	- (-,-)
Vitamin C	2 (1.7)	4 (0.59)	0.15	3.77 (0.48,21.86)	-	-	-	-
Vitamin D	117(100 %)	680 (100%)	-	- (-,-)	-	-	-	- (-,-)
Vitamin E	-	-	-	- (-,-)	-	-	-	- (-,-)
Vitamin K as phylloquine	0 (0%)	6 (0.882%)	0.98	$1.61*10^{-6}$ (-,$1.67*10^{18}$)	-	-	-	-
**Minerals**								
Calcium	-	-	-	- (-,-)	-	3 (0.44)	0.98	$2.26*10^{-6}$ (-,$6.81*10^{22}$)
Copper	1 (0.85)	8 (1.18)	0.83	0.78 (0.04,4.74)	-	-	-	-
Iodine	-	7 (1.03)	0.98	$1.38*10^{-6}$ (-,$1.46*10^{15}$)	-	-	-	- (-,-)
Iron	-	1 (0.15)	0.98	$7.98*10^{-6}$ (-,$1.91*10^{42}$)	-	-	-	-
Magnesium	-	3 (0.44)	0.98	$2.77*10^{-6}$ (-,$1.82*10^{23}$)	21 (17.9)	90 (13.24)	0.52	1.22 (0.64,2.26)
Manganese	-	-	-	- (-,-)	-	-	-	-
Phosphorus	-	2 (0.3)	0.98	$2.73*10^{-6}$ (-,$8.15*10^{36}$)	-	-	-	-
Potassium	-	3 (0.44)	0.98	$2.77*10^{-6}$ (-,$1.82*10^{23}$)	-	-	-	-

Sodium	-	7 (1.03)	0.98	$1.48*10^{-6}$ (-,$9.98*10^{14}$)	-	-	-	-
Zinc	-	2 (0.3)	0.98	$2.73*10^{-6}$ (-,$8.15*10^{36}$)	-	1 (0.15)	0.98	$8.44*10^{-7}$ (-,$1.34*10^{41}$)
**Water**			0.98	$2.73*10^{-6}$ (-,$1.5*10^{23}$)	-	-	-	-
**Alcohol**	-	-	-	- (-,-)	-	-	-	-

"-" means there were not enough data to draw conclusions

Table S5. Intakes of micronutrients below 2SDs or above the Upper level (UL) as suggested by the European Food and Safety Authorities (EFSA) for pregnant women, during pregnancy.

Micronutrients	Until first half of gestation							
	Intakes below 2SDs				Intakes above the UL			
	GDM (N%)	Non GDM (N%)	p value	aOR 95% CI	GDM	Non GDM	p value	aOR 95% CI
**Vitamins**								
Biotin	-	1 (0.15)	0.98	$9.96*10^{-6}$ (-,$2.23*10^{42}$)	-	-	-	-
Cobalamin (Vitamin B12)	1 (0.85)	3 (0.44)	0.41	2.69 (0.12,22.8)	-	-	-	-
Folate	1 (0.85)	7 (1.03)	0.89	0.86 (0.04,5.41)	-	-	-	- (-,-)
Niacin			0.2	6.46 (0.24,174.97)	-	-	-	-
Pantothenic acid	1 (0.85)	4 (0.59)	0.71	1.53 (0.07,11.26)	-	-	-	-
Riboflavin (Vitamin B2)	1 (0.855%)	2 (0.294%)	0.3	3.76 (0.16,42.66)	-	-	-	-
Thiamin (Vitamin B1)	1 (0.855%)	1 (0.147%)	0.2	6.46 (0.24,174.97)	-	-	-	-
Vitamin A	2 (1.71%)	16 (2.35%)	0.75	0.78 (0.12,2.93)	-	-	-	- (-,-)
Vitamin B6	1 (0.85)	3 (0.44)	0.61	1.83 (0.08,15.42)	-	-	-	- (-,-)
Vitamin C	-	4 (0.59)	0.98	$9.46*10^{-7}$ (-,$9.35*10^{29}$)	-	-	-	-
Vitamin D	117 (100%)	680 (100%)	-	- (-,-)	-	-	-	- (-,-)
Vitamin E			0.98	$3.04*10^{-6}$ (-,$7.52*10^{36}$)	-	-	-	- (-,-)
Vitamin K as phylloquine	1 (0.855%)	9 (1.32%)	0.71	0.67 (0.03,3.82)	-	-	-	-
**Minerals**								
Calcium	1 (0.85)	1 (0.15)	0.2	6.46 (0.24,174.97)	1 (0.85)	1 (0.15)	0.5	2.72 (0.09,75.29)
Copper	1 (0.85)	8 (1.18)	0.81	0.76 (0.04,4.51)	-	-	-	-
Iodine	2 (1.7)	7 (1.03)	0.44	1.91 (0.27,8.56)	-	-	-	- (-,-)
Iron	2 (1.7)	4 (0.59)	0.3	2.62 (0.33,15.07)	-	-	-	-
Magnesium	1 (0.85)	4 (0.59)	0.75	1.43 (0.07,10.53)	26 (22.2)	88 (12.9)	0.066	1.71 (0.95,3)
Manganese	-	-	-	- (-,-)	-	-	-	-

Phosphorus	2 (1.7)	5 (0.74)	0.3	2.47 (0.33,12.54)	-	-	-	-
Potassium	1 (0.85)	5 (0.74)	0.8	1.34 (0.06,9.32)	-	-	-	-
Sodium	1 (0.85)	5 (0.74)	0.98	0.96 (0.04,6.63)	-	-	-	-
Zinc	1 (0.855%)	6 (0.882%)	0.9	1.14 (0.05,7.37)	-	-	-	- (-,-)
**Water**	2 (1.71%)	6 (0.882%)	-0.5	1.76 (0.24,8.14)	-	-	-	-
**Alcohol**	-	-	-	- (-,-)	-	-	-	-

"-" means there were not enough data to draw conclusions

Table S6. Adherence to MDS and risk for GDM before and during pregnancy for the Mean adequacy ratio (MAR).

Adherence to Mediterranean Diet (Score)				
	LOW (0-5)		High (6-9)	
**Before pregnancy**	p-value (aOR)	aOR (95% CI)	p-value (aOR)	aOR (95% CI)
MAR	0.84	0.99 (0.98,1.01)	0.75	0.99 (0.98,1.01)
MAR reduced	0.74	0.91 (0.54,1.52)	0.75	0.89 (0.44,1.74)
**During pregnancy**				
MAR	0.61	0.99 (0.97,1.01)	0.23	1.01 (0.99,1.03)
MAR reduced	0.68	0.83 (0.33,1.95)	0.29	1.66 (0.63,4.35)

MDS: Mediterranean diet score; GDM: gestational diabetes mellitus; MAR: mean adequacy ratio; MDS scoring scale by Trichopoulou et al. The adjusted Odds Ratio (aOR) was computed using a logistic regression model. The adjustment was made with respect to: energy, ART, supplements intake, physical activity, BMI, maternal age, thyroid status, smoking status, and parity status.

Figures

Figure S1. Vitamin intakes above EFSA recommendations in GDM and non-GDM groups for up to six months before pregnancy.

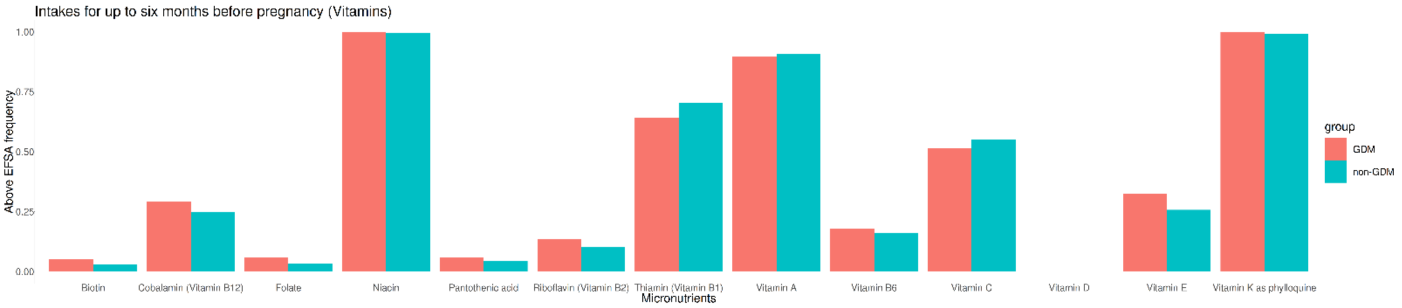

Figure S2. Mineral and water intakes above EFSA recommendations in GDM and non-GDM groups for up to six months before pregnancy.

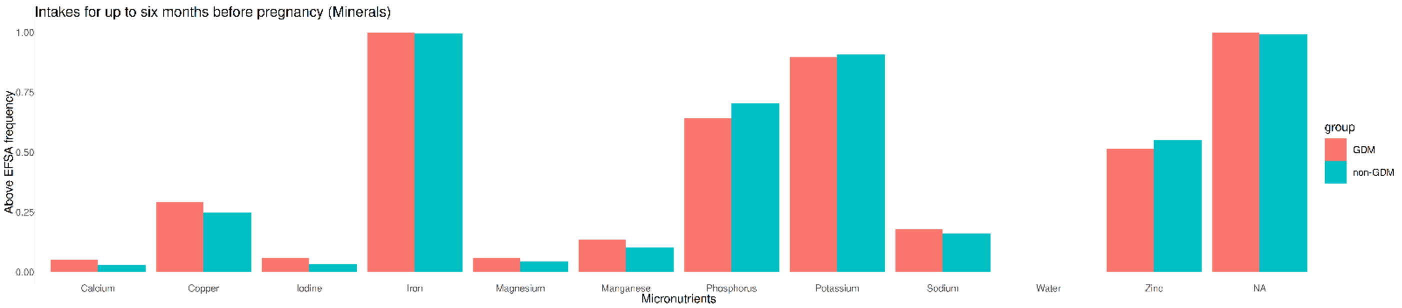

Figure S3. Vitamin intakes above EFSA recommendations in GDM and non-GDM groups during pregnancy.

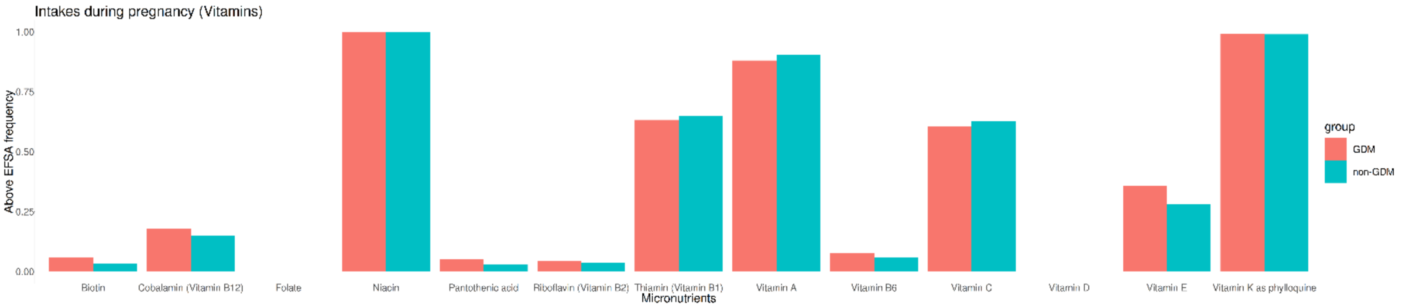

Figure S4. Mineral and water intakes above EFSA recommendations in GDM and non-GDM groups during pregnancy.

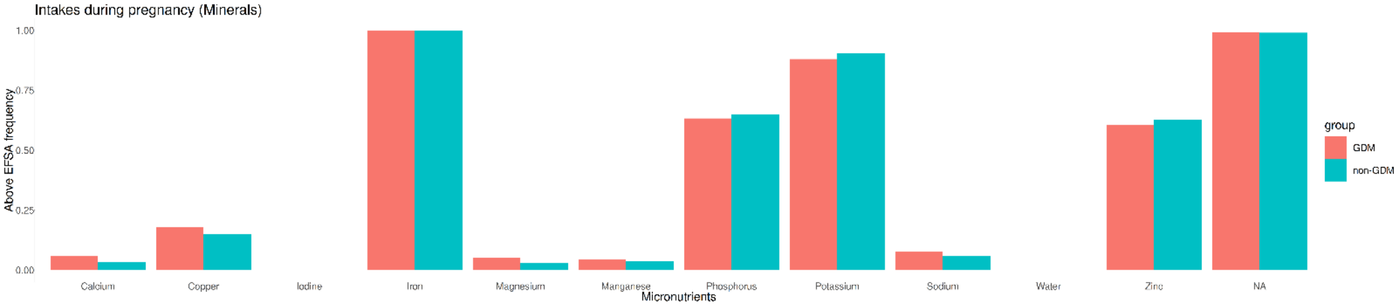

Supplement: Supplementary file 1 [file nutrients-16-01375-s001.zip › nutrients-2950385-supplementary.pdf]
